# Neutralizing Dengue Antibody in Pregnant Thai Women and Cord Blood

**DOI:** 10.1371/journal.pntd.0003396

**Published:** 2015-02-06

**Authors:** Kriangsak Khamim, Weerawan Hattasingh, Ananda Nisalak, Jaranit Kaewkungwal, Stefan Fernandez, Butsaya Thaisomboonsuk, Krisana Pengsaa, Usa Thisyakorn

**Affiliations:** 1 Department of Obstetrics and Gynecology, Ban Pong Hospital, Ratchaburi, Thailand; 2 Department of Tropical Pediatrics, Faculty of Tropical Medicine, Mahidol University, Bangkok, Thailand; 3 Department of Virology, Armed Forces Research Institute of Medical Sciences (AFRIMS), Thailand; 4 Centre of Excellence for Biomedical & Public Health Informatics (BIOPHICS), and Department of Tropical Hygiene, Faculty of Tropical Medicine, Mahidol University, Bangkok, Thailand; 5 Department of Pediatrics, Faculty of Medicine, Chulalongkorn University, Bangkok, Thailand; Institute of Tropical Medicine (NEKKEN), JAPAN

## Abstract

**Background:**

The WHO ‘Global Strategy for Dengue Prevention and Control, 2012–2020’ addresses the growing need for the treatment of dengue, and targets a 25% reduction in morbidity and 50% in mortality (using 2010 estimates as baseline). Achieving these goals requires future dengue prevention strategies that will employ both potential vaccines and sustainable vector-control measures. Maternally transferred dengue antibody is an important factor in determining the optimal age for dengue vaccination.

**Objectives:**

To estimate the seroprevalence of dengue antibodies among mothers living in an area of high endemicity – Ban Pong, Ratchaburi Province – and to assess maternal dengue antibodies transferred to cord blood.

**Materials & Methods:**

A cross-sectional study was conducted with 141 pregnant women who delivered at Ban Pong Hospital, Ratchaburi, Thailand. Maternal-cord paired sera were tested for dengue neutralizing (NT) antibody by PRNT_50_ assay. A ratio of ≥ 1:10 NT titer to dengue serotype was considered seropositive.

**Results:**

Most mothers (137/141, 97.2%) had NT antibodies to at least one dengue serotype in their sera. At birth, the proportion of cord sera with NT antibodies to DEN-1, DEN-2, DEN-3, and DEN-4, were high and similar to the sera of their mothers, at 93.6%, 97.2%, 97.9%, and 92.2%, respectively. The dengue geometric mean titers (GMT) in cord blood were significantly higher than the maternal antibodies (p<0.001): highest in DEN-2, followed by DEN-3, and then DEN-1. The GMT of DEN-4 was the lowest among all four serotypes.

**Conclusions:**

Dengue infection is highly prevalent among pregnant women in this dengue-endemic area. Most of the cord blood had transferred dengue antibodies, which may have an impact on the disease burden in this population.

## Author Summary

Dengue is the fastest spreading mosquito-borne viral infection. Infections cause mild to severe diseases, including dengue hemorrhagic fever (DHF), a severe form that may kill infants and young children. Dengue virus antibody transfer from mother to fetus in pregnancy confers protection at birth, thereafter subsiding to a lower level that may cause DHF in infants. Infant dengue antibodies levels also influence the optimal age for dengue vaccination because of neutralization of the proposed live virus vaccine by the protective antibody levels in the newborn. To establish the optimal age, we identified mother-child pairs in which maternal dengue antibodies were transferred from mother to fetus in this study. Then a follow-up study would measure the infant antibody levels. Our study found that 97.2% of pregnant women giving birth in a dengue-endemic area had evidence of previous dengue infection. All umbilical cord blood from fetuses had the same proportion of positive tests for the presence of dengue antibodies, but had a higher dengue antibody levels compared to their mothers. The period of protection provided by maternally transferred dengue antibodies might affect the disease burden among infants and offer a better understanding of the optimal age for dengue vaccination.

## Introduction

Dengue is the most rapidly disseminating mosquito-borne viral infection [1]. Any of the 4 antigenically-related serotypes DEN-1, DEN-2, DEN-3 or DEN-4 may cause an infection with a wide variety of manifestations from mild to severe such as asymptomatic infection, undifferentiated febrile illness, dengue and severe dengue infection [2]. The pathological processes of the severe forms of infection, including dengue hemorrhagic fever (DHF), remain unclear. Because cases of dengue virus infected infants <1 year old with maternal dengue virus antibodies at a subneutralizing level have shown a greater probability of contracting DHF, antibody-dependent enhancement (ADE) has been suggested as a possible process [3–6].

Having been first detected in hospitalized Thai patients in Bangkok in 1958 [7], dengue virus infections have occurred in other regions of the country [8, 9]. The majority of dengue infections in Asia are in children, and it is one of the 10 most common causes of morbidity and mortality for children in the region [9]. An initial estimate of up to 3.97 billion people might be at risk of infection [10]. Of an estimated 390 million dengue infections, 96 million have clinical manifestations. This is just over four times that of the dengue burden estimated by the World Health Organization (WHO) [11]. Globally, Asia, the Americas and some Pacific islands have had dengue epidemics. The majority of epidemics (75%) occur in the WHO defined regions of Southeast Asia (SEA) and the Western Pacific [2]. With the exception of the Maldives, Nepal and Thailand, other SEA countries reported increasing numbers of cases between 2011 and 2012 [12].

The only immunological substance recognized to be transferred from mother to fetus are antibodies, of which most are in the IgG subclass [13–16]. Measurements of high levels of transferred neutralizing dengue antibodies have been found in neonates at delivery [17, 18]. Proven by serum sampling, the presence of these antibodies in SEA region infants prevents clinical dengue before around 6–9 months of age [9, 17–19].

To address the increasing need for dengue treatment, the WHO’s ‘Global Strategy for Dengue Prevention and Control, 2012–2020’ set targets to reduce dengue morbidity by 25% and mortality by 50% (calculated from a baseline of the 2010 estimate) by 2020 [1, 20]. To reach these targets, the use of potential vaccines and sustainable vector control measures are the main prevention strategies, as well as appropriate clinical management. At the time of writing, no licensed vaccine exists. Recombinant live-attenuated, CYD tetravalent dengue vaccine was reported to provide around 30% effective protection in a trial of Thai school children [21]. In Asia and Latin America, a multicentered phase III trial has been ongoing since 2011. In Thailand, this trial involves Ban Pong and Photharam Districts in Ratchaburi Province and Kamphaeng Phet Province. One of the top 10 provinces in incidence of dengue in Thailand, Ratchaburi Province is situated 100 km west of Bangkok. The incidence of clinical dengue rose from 123.45 per 100,000 population in 2003 to 394.25 in 2008 [22].

One of two key Phase III efficacy studies of a potential dengue vaccine has successfully attained its primary clinical endpoint. From a preliminary press release, this study reported a 56% decrease in dengue disease cases, and preliminary safety data suggests a good safety profile similar to previous studies [23]. To decide future vaccination strategies, accurate data of measurements of disease impact and of the kinetics of maternal dengue antibodies are crucial.

We designed this study to evaluate the seroprevalence of neutralizing dengue antibody of pregnant women living in an area of high endemicity of dengue—Ban Pong, Ratchaburi Province—and to ascertain the proportion of transference of maternal neutralizing dengue antibody to the cord blood.

## Materials and Methods

### Setting

Ban Pong Hospital, a 350-bed state-operated hospital, services a district of 169,900 people and an average of 160 babies are delivered there every month.

### Study design

At Bang Pong Hospital, we conducted a prospective observational cross-sectional study and enrolled 151 mother-infant pairs between December, 2011 and January, 2013. We excluded pregnant women who had a history of immunodeficiency, had taken immunosuppressive medications within a month before the study (inclusive of corticosteroid treatment >2 weeks), and had been transfused blood or blood components within 3 months before the study.

### Sample size calculation

We determined the sample size to evaluate the seroprevalence of dengue infection in pregnant women by Epi Info software (2002). The basis of our calculation was a survey of pregnant women in Bangkok, Thailand from 2000 to 2001. In a sample size of 124, that survey used a 50% plaque reduction neutralization test (PRNT_50_), and reported a prevalence of dengue antibody at 97% [20] with a confidence interval of 95% and a precision of +/- 3%. From this basis, we derived a total sample size of 150 pregnant women.

### Data collection

We noted the demographic data of the mothers and neonates within 24 hours after delivery. We collected a 5-ml blood sample from each mother and a 5-ml sample of cord blood at delivery for dengue serological testing.

### Serology

Every sample was centrifuged within several hours after collection and stored at -20°C. The Armed Forces Research Institute of Medical Sciences (AFRIMS), Bangkok, Thailand performed the antibody assays.

Dengue antibody levels against all 4 dengue reference strains were performed by PRNT_50_ technique previously described by Russell et al [24] in 141 dengue hemagglutination assay seropositive maternal sera. The dengue strains used were DEN-1(16007) (isolated from a child with DHF in Thailand in 1964), DEN-2 (16681) (isolated from a child with DHF in Thailand in 1964), DEN-3 (16562) (from a child with DHF in the Philippines in 1964) and DEN-4 (C0036/06) (from a child with DF in Thailand in 2006). PRNT titer ≥1:10 to one dengue serotype at least was considered seropositive.

### Data analysis

We calculated serotype-specific seroprevalence rates to be the percentage of samples with neutralizing (NT) antibody levels ≥1:10 against at least one dengue serotype. We calculated the geometric mean titers (GMTs) at a 95% confidence interval (CI) for each of the 4 serotypes. The comparisons of seroprevalence and GMTs between mother and paired cord blood were performed by symmetry test and paired t-test, respectively. Comparisons between seroprevalences among different age specific groups were based on chi-square test. Correlations between maternal and cord blood of DEN-1, DEN-2, DEN-3 and DEN-4 antibody titers were computed by Spearman’s rho. The paired titers of mothers and cord bloods were also plotted using lowess smoothing curve. All statistical analyses were performed using STATA software (version 10). The level of significance was set at 0.05.

### Ethics

The Ethics Committee of Ban Pong Hospital approved the study protocol. All the pregnant women gave written informed consent prior to enrollment.

## Results

We enrolled 141 mothers with a mean (± SD) maternal age of 23.6 years (± 5.8 years; range 15–41 years). All participants resided in Bang Pong District, Ratchaburi Province.

Just over one-third of the mothers were <20 years of age. Slightly less than 44% of them were primigravida. Table 1 displays the demographic data of the mothers including age, level of education, occupation, number of parity, gestational age and mode of delivery. All neonates were healthy at birth. Two had low birth weights (<2,500 grams) at 2,190 grams and 2,460 grams. A proportion of 99.3% were full term. Mean birthweight (± SD) was 3,196 (± 399) grams (range 2,190–4,430 grams). The male to female ratio was 1.1:1.

**Table 1:** Demographic characteristics of 141 pregnant women.

Variable	Number (%)
Age	
- ≤20 y	54 (38.3)
- 21–25 y	40 (28.4)
- 26–30 y	20 (14.2)
- 31–35 y	24 (17.0)
- ≥36 y	3 (2.1)
Education
- primary school	58 (41.1)
- secondary school	66 (46.8)
- vocational school	11 (7.8)
- bachelor degree	6 (4.3)
Occupation	
- housewives	67 (47.5)
- employee	54 (38.3)
- other	20 (13.2)
Parity	
- 0	61 (43.3)
- 1	51 (36.2)
- >2	29 (20.5)
Gestational age (wks)	
- 37–41	140 (99.3)
- ≥42	1 (0.7)
Mode of delivery	
- spontaneous delivery	134 (95.0)
- vacuum extraction	2 (1.4)
- cesarean section	5 (3.6)

Some factors suspected to influence the prevalence of dengue infection such as age, level of education, occupation and the household income of the mothers were analyzed by logistic regression. These analyses produced no statistically significant differences.

**Fig 1:**
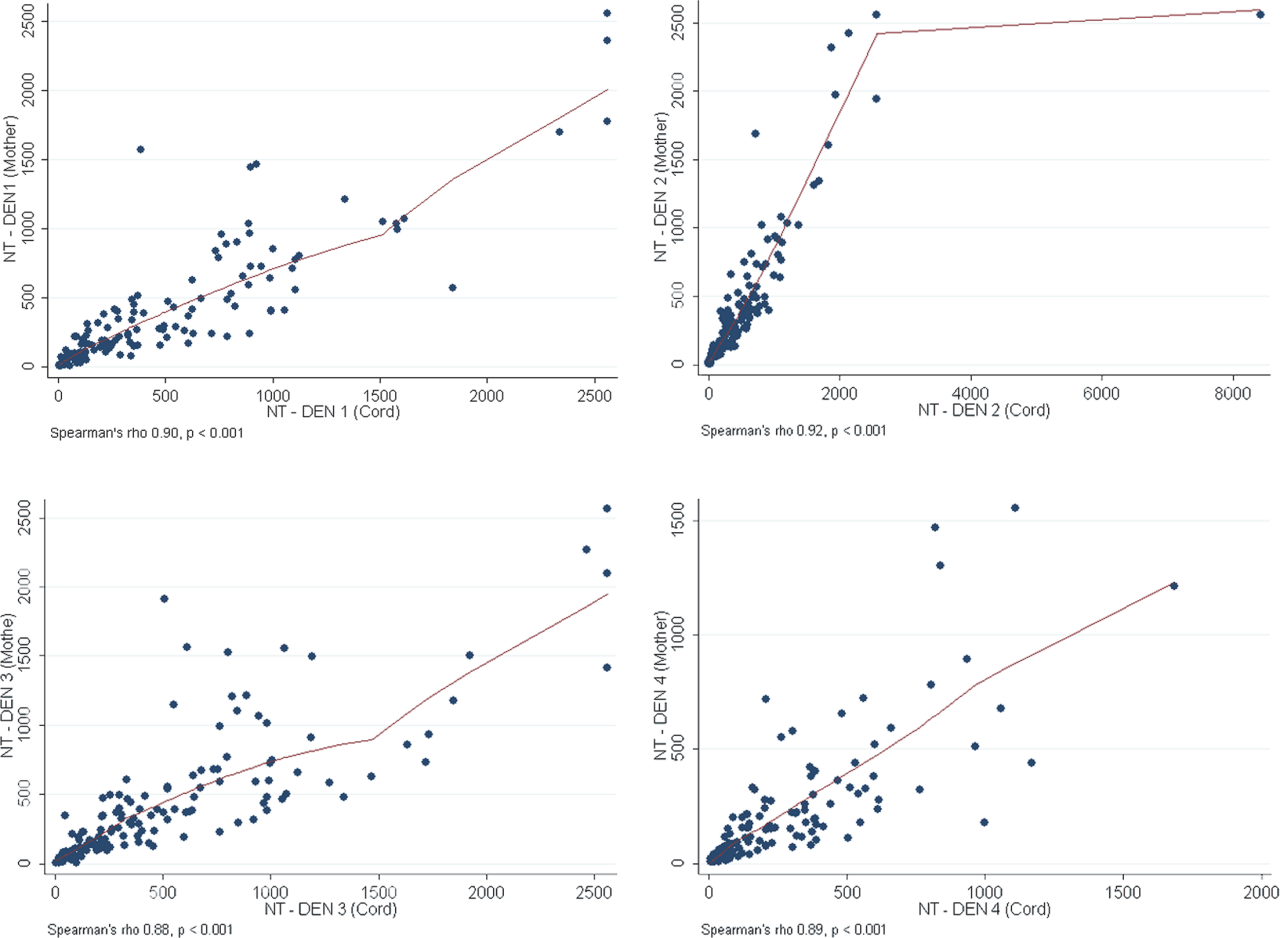
Correlation between maternal and cord blood of DEN-1 to DEN-4 neutralizing antibodies titers. X-axis shows GMTs of cord blood dengue antibody and Y-axis shows GMTs of maternal dengue antibody of DEN-1, DEN-2, DEN-3 and DEN-4. The Spearman’s rho of DEN1, DEN2, DEN3 and DEN4 are 0.90, 0.92, 0.88 and 0.89 respectively.

Most mothers (137/141, 97.2%) had NT antibody to at least 1 dengue serotype in their sera. Table 2 displays the proportions of seroprevalence of all 4 dengue serotypes, and GMT levels of the pregnant women and their cord sera. At birth, the proportion of cord sera with NT antibodies to DEN-1, DEN-2, DEN-3 and DEN-4 were high and similar to the sera of their mothers at 93.6, 97.2, 97.9 and 92.2%, respectively. The GMT levels of dengue NT titers in cord blood were significantly higher than the maternal antibody titers (p<0.001): the highest in DEN-2, followed by DEN-3, and DEN-1. The GMT of DEN-4 was the lowest of all the 4 serotypes. Maternal and cord NT of DEN-1, DEN-2, DEN-3, and DEN-4 antibody titers correlated well (Spearman correlation <0.001), as shown in Fig. 1.

**Table 2:** The proportion and geometric mean titer of NT dengue antibodies of 141 maternal and cord paired sera.

		Positive (N)	%	GMTs	(95%CI)
DEN-1	mother	132	93.6	180.3	141.5–229.7
	Cord	132	93.6	219.0	169.6–282.9
	p-value	1.00		<0.001	
DEN-2	mother	137	97.2	246.0	201.0–301.1
	Cord	137	97.2	308.1	249.7–380.1
	p-value	1.00		<0.001	
DEN-3	mother	136	96.5	233.9	187.1–292.4
	Cord	138	97.9	288.1	232.0–357.6
	p-value	0.157		<0.001	
DEN-4	mother	127	90.1	90.3	71.0–114.8
	Cord	130	92.2	120.7	95.2–152.9
	p-value	0.180		<0.001	
					

Note: p-values for comparisons of seroprevalence are based on symmetry test; p-values for comparisons of GMTs between mother and cord are based on paired test of log (NT)

All mothers aged >35 years and their cord sera showed 100% seropositive to all 4 dengue serotypes compared with the younger age groups. However, dengue seroprevalence by maternal age grouping was not statistically significantly different.

## Discussion

In our study, we found that 97.2% of pregnant women giving birth at Ban Pong Hospital showed serological evidence of previous dengue infection. Most transferred dengue antibodies to cord blood. The high levels of antibody transference at delivery in our study are similar to previous results from Bangkok, Thailand, from 1998 to 2001 [17,18, 25,26]. The high proportion of mothers with antibodies against multiple dengue virus serotypes reflects the high rate of transmission of dengue viruses in an endemic area, Thailand.

Seronegative sera occurred in 2.8% of pregnant women possibly susceptible to primary dengue infection, and another 2.8% (4/141) would be at risk to secondary dengue infection because they had only one dengue serotype positivity.

A detectable dengue IgG of 53.9% in the pregnant women using dengue IgG indirect enzyme-link immunosorbent assay (ELISA) with a high degree of agreement of IgG seropositivity between the pairs of pregnant women and their neonates (99.3%) was reported in a cross-sectional study in central Brazil during a large outbreak (2009–2010) [27]. Approximately half of the adult population screened was still immunologically naïve to dengue virus exposure. This finding appears compatible with the more recent reintroduction of the virus to central Brazil.

Table 3 shows a comparison of maternal dengue seroprevalence, maternal and cord sera antibody titers in various published articles [17, 18, 25–28]. However, the dengue seroprevalence of IgG in pregnant women in previous reports detected by ELISA IgG was 93.6–95.2% [17, 18]. Our finding that maternally transferred neutralizing dengue antibody in cord blood in all 4 dengue serotypes had a higher GMT than maternal blood with statistically significant differences (p <0.001) is consistent with previous reports [13, 25]. Higher cord blood GMTs compared to maternal blood in all 4 dengue serotypes using HAI assay were reported [26] although only 52.9% of mean dengue antibody was higher in cord sera than maternal sera [25]. Only higher GMTs of DEN-2 in cord sera compared to maternal sera were found while GMTs of DEN-1, DEN-3 and DEN-4 were higher in maternal sera compared to cord sera [18]. This could be explained by the preferential movement of the antibody across the placenta, which promotes both greater avidity to antigens and greater ease of transplacental passage, and reflects the dynamic of IgG placental transfer [13, 28]. Most infants experience a decline in maternal antibody level during the first 12 months [17, 18, 25, 26], and some infants are at risk of developing various spectra of dengue infection [18]. Undifferentiated fever and asymptomatic dengue infection have been diagnosed in infants after a decline of maternal-transferred antibody [18, 19]. Because of this, refinement and reconsideration of the current ADE pathogenesis should be encouraged [19].

**Table 3:** Comparison of maternal dengue seroprevalence, maternal and cord sera dengue antibody titers in various articles.

	Methods	Seroprevalence (%)	Maternal GMTs	Cord GMTs	p*
Pengsaa K, et al[17]	PRNT_50_	95.2	-	53.5–258.2^a^	n/a
Watanaveeradej V, et al[25]	HAI	96.8	-	-	n/a
	IgG	-	62.7±85.8^b^	99.8±448^b^	n.d.
Perret C, et al[26]	HAI	94.7	41.3–104^a^	50.8–128.2^a^	<0.01^d^
Pengsaa K, et al[18]	PRNT_50_	97.3	82.1–731^a^	78.6–692^a^	n.d.
Chau TNB, et al[28]	PRNT_50,_	98	26–53^a^913 (582–1,297)^c^	30–80^a^1,025 (600–1,429)^c^	<0.001^e^
	IgG		913 (582–1,297)^c^	1,025 (600–1,429)^c^	0.002
Argolo AF, et al[27]	IgG	53.9	-	-	n/a
This study	PRNT_50_	97.2	90.3–246^a^	120.7–308.1^a^	<0.001

* p-values are based on paired sample t-test

^a^ range

^b^ mean±SD

^c^ mg/dL, GMT (range)

^d^ p-value 0.002 for DEN-1, 0.003 for DEN-3 and <0.001 for DEN-2 and DEN-4

^e^ p-value <0.001 for DEN-2 and DEN-3 only, p-value for DEN-1 and DEN-4 ≥ 0.5

*n/a* = not applicable, *n*.*d*. *=* no data available

Epidemiological, clinical, and virological studies indicate that the disease severity could be due to viral virulence, as well as the presence of passively acquired heterotypic sub-neutralizing maternally-transferred dengue antibodies which cause ADE [3–6].

In our study, we demonstrated that the seroprevalence of dengue infection in pregnant women to all 4 dengue serotypes was the highest in DEN-2, followed by DEN-3, DEN-1 and DEN-4. These finding agree with the high incidence of dengue infection with all 4 dengue serotypes in primary school children in the Namuang Subdistrict of Muang District, Ratchaburi Province from 2006 through 2009 [29]. However, the rank order of seroprevalence in that study was dissimilar to that of the pregnant women in our study. DEN-1 was the most common infecting serotype reported in those school children (43%), followed by DEN-2 (29%), DEN-3 (20%) and DEN-4 (8%). These findings indicate that dengue transmission is continuous in Ratchaburi Province.

The high dengue seroprevalence in older mothers in our study is similar to a previous study in Bangkok in which all mothers aged >35 years transferred antibodies to their infants [25]. The higher prevalence of dengue infection among older mothers compared to younger mothers reflects the high transmission rate of dengue viruses in Thailand, and confirms the findings of previous studies [18, 25, 26].

### Conclusion

A high neutralizing dengue seroprevalence of 97.2% was found in pregnant women at Ban Pong Hospital, Ratchaburi Province. Nearly all those pregnant women had been infected with the 4 dengue serotypes. All cord blood had the same proportion of seropositivity, but had a higher dengue antibody titer to each dengue serotype compared to their mothers. Most infants appeared to be protected from dengue infection in early life by maternal transferred dengue antibodies. In this dengue-endemic area, the period of protection provided by maternally transferred dengue antibodies might have an impact on the disease burden among infants, and offer a better understanding of the optimal age for dengue vaccination.

**Disclaimer**: The views expressed in this article are those of the author (s) and do not reflect the official policy of the Department of the Army, Department of Defense, or the U.S. Government.

## Supporting Information

S1 Checklist STROBE Checklist. (DOC)
